# High Strain Survivability of Piezoceramics by Optimal Bonding Adhesive Design

**DOI:** 10.3390/s18082554

**Published:** 2018-08-04

**Authors:** Hu Sun, Yishou Wang, Xinlin Qing, Zhanjun Wu

**Affiliations:** 1 School of Aerospace Engineering, Xiamen University, Xiamen 361005, China; sunhu@xmu.edu.cn (H.S.); wangys@xmu.edu.cn (Y.W.); 2 School of Aeronautics and Astronautics, Dalian University of Technology, Dalian 116024, China; wuzhj@dlut.edu.cn

**Keywords:** adhesive, piezoceramic, sensor, strain survivability, structural health monitoring

## Abstract

As one of the most common transducers used in structural health monitoring (SHM), piezoceramic sensors can play an important role in both damage detection and impact monitoring. However, the low tensile strain survivability of piezoceramics resulting from the material nature significantly limits their application on SHM in the aerospace industry. This paper proposes a novel approach to greatly improve the strain survivability of piezoceramics by optimal design of the adhesive used to bond them to the host structure. Theoretical model for determining the strain transfer coefficient through bonded adhesive from the host structure to piezoceramic is first established. Finite element analysis is then utilized to study the parameters of adhesive, including thickness and shear modulus. Experiments are finally conducted to validate the proposed method, and results show the piezoceramic sensors still work well when they are bonded on the host structures with tensile strain up to 4000 με by using the optimal adhesive.

## 1.Introduction

Structural health monitoring (SHM) technology is perceived as a revolutionary method of determining the integrity of structures, involving the use of multi-disciplinary fields including sensors, materials, signal processing, system integration, and signal interpretation [1,2,3]. Among several types of sensors that can be used for SHM, piezoceramic lead-zirconate-titanate (PZT) sensors have big potential for being widely used because they can be used for both active and passive monitoring. In active model, guided wave theory and electromechanical impedance theory are often used [4,5,6,7,8,9]. Different from conventional ultrasonic technique, the PZT sensors for SHM are permanently bonded on their host structure. Because of small additional weight and high sensitivity to small damage, PZT sensors with thin thickness, which show d31-type piezoelectric effect, are often used in the SHM application for aircraft structures [10,11].

The reliability of PZT sensor network for SHM must be entirely guaranteed through the life of host structures. However, the environmental features, such as mechanical loading, temperature, and humidity, can significantly degrade the performance of PZT sensors due to the nature of the PZT material. In particular, PZT sensors can only theoretically survive low tensile strain of 1100 με, which is usually much lower than the strain level of aircraft structures. Some studies were conducted to investigate the durability and survivability of PZT sensors, and different results were obtained when the PZT sensors were exposed to various experimental conditions. For example, one showed that PZT remained operational up to at least 3000 με and failed beyond 6000 με during high-strain tests [12], but another study showed that the amplitude of the guided wave received by a PZT sensor remained unchanged when the applied strain was lessr than the PZT elongation value of about 1100 με, whereas degradation occurred when the strain exceeded it [13]. These different results could be caused from the effect of adhesive used to bond the PZT sensor.

Typically, the PZT sensor network used for SHM is permanently bonded with adhesive to the host structure. The adhesive forms an interfacial layer of finite thickness between the piezoelectric element and host structure, and provides the necessary mechanical coupling needed to transfer the forces and strains between the piezoelectric element and the host structure [12]. The effect of adhesive thickness and its modulus on the performance of adhesively bonded PZT sensors for the purpose of monitoring structural health has been investigated experimentally [14]. The experimental results revealed that an increase in adhesive thickness alters the electromechanical impedance and the resonant frequency of the PZT sensor as well as the amplitude of the sensor signal. When the modulus is within a certain range, the modulus of the adhesive slightly affects the impedance of the PZT sensor and the amplitude of lower frequency sensor signals, while at high frequency, the impedance response and sensor signal are more sensitive to the modulus of the adhesive. The influence of the adhesive layer on the electromechanical impedance of PZT sensor used has been studied by a few researchers [15,16,17]. Similarly, for guided wave applications, assessment and selection of five types of adhesive were experimentally investigated by Mustapha and Ye [18]. A hybrid spectral element method was adopted to analyze the influence of adhesive thickness and stiffness, and PZT diameter and thickness on the guided wave generation and reception [19]. The effect of adhesive, the tone burst, the plate thickness and the transducer thickness on the time reversibility of Lamb waves was also investigated by Agrahari et al [20]. In addition, a compensation method of the degradation of the adhesive layer for damage detection in SHM was proposed by Mulligan [21].

Even though many simulation and experimental works have been conducted to investigate the effect of adhesive on the functions of PZT sensors, there is little work performed to study the effect of adhesives on strain transfer from the host structure to the PZT, which would provide a strategy to significantly increase the high tensile strain-bearing ability of PZT sensors to ensure the durability and reliability of SHM systems in real world applications. A preliminary experimental study showed that the high-strain survivability ability of PZT can be enhanced by using different the adhesive material [22]. In this paper, a further study containing analytical, finite element and experimental solution was conducted to give a systematic understanding about how to increase the high tensile strain bearing ability of PZT. A theoretical model of strain transfer through the adhesive from the host structure to the PZT was first proposed. Then finite element simulation, as well as analytical solution, were conducted to analyze the effect of the adhesive parameters on the strain transfer, which would be used to select a suitable adhesive to reduce the strain of the PZT. Experimental research was finally conducted to validate the analytical and finite element results, which reveal that reducing the shear modulus and enhancing the thickness of the adhesive can decrease the strain transfer from the host structure to the PZT because of the shear lag effect.

## 2. Theoretical Model of Strain Transfer

**Figure 1:**
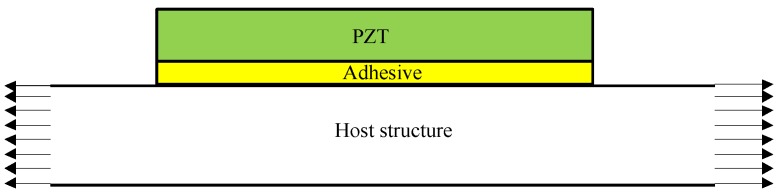
Theoretical model of strain transfer among PZT-adhesive-host structure.

For a PZT plate bonded on a host structure shown in Figure 1, there are three basic components, i.e., PZT, adhesive layer and host structure. The strain of the host structure is transferred to the PZT by the adhesive layer.

The problem of bonding between PZT sensor and host structure is three-dimensional problem. However, this three-dimensional problem is very difficult to carry out accurate mathematical analysis. Through some reasonable simplification, the problem can be simplified to one-dimension problem, and only the stress and strain of the whole structure in the direction of tension are considered since the maximum strain direction on the PZT sensor is basically the same as the tensile direction.

Using the theoretical uniform strain model proposed by Crawley [23,24,25] as the basis, the model of strain transfer between the PZT and the host structure can be obtained based on some assumptions as follows:

(1) The adhesive transfers shear stress between the PZT and the host structure;
(2) The PZT is uniformly extended;
(3) Extension strain ε is loaded at two ends of the host structure, while two ends of PZT are free.

**Figure 2:**
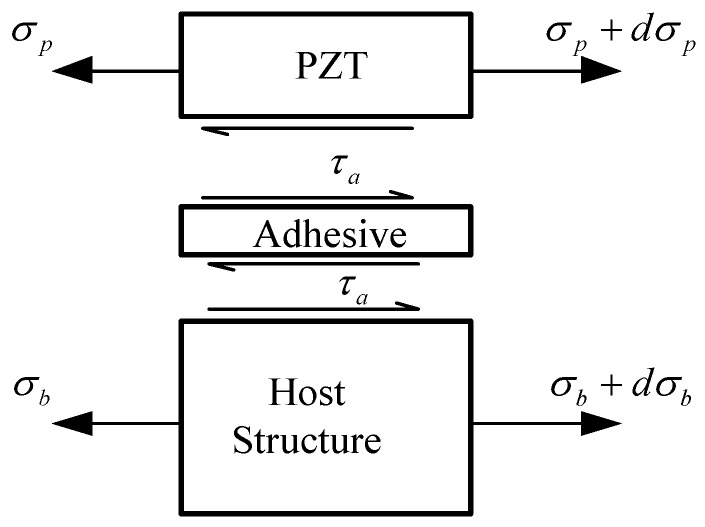
The force equilibrium of the micro structural element.

Considering a micro element of the host structure bonded with the PZT through the adhesive, it is loaded as shown in Figure 2. The equilibrium equations of PZT and the host structure can be written as:

(1) $$\frac{d\sigma_{p}}{dx}-\frac{\tau_{a}}{h_{p}}=0,\frac{d\sigma_{b}}{dx}+\frac{\alpha \tau_{a}}{h_{b}}=0$$

where *h_p_* and *h_b_* are the thickness of the PZT and the host structure, respectively. Parameter α depends on the stress and strain distributions of the host structure along the thickness direction. At the low frequency range, α=1 if the host structure is purely extended. α=3 when the host structure is purely bended. α=4 for the axial-bending interaction status. 

The strain of the PZT, the host structure and the adhesive can be derived as:

(2) $$\varepsilon_{p}=\frac{du_{p}}{dx},\varepsilon_{b}=\frac{du_{b}}{dx},\gamma_{a}=\frac{u_{p}-u_{b}}{h_{a}}$$

where *h_a_* is the adhesive thickness.

The stress of the PZT, the host structure and the adhesive can be obtained as:

(3) $$\sigma_{p}=E_{p}\varepsilon_{p},\sigma_{b}=E_{b}\varepsilon_{b},\tau_{a}=G_{a}\gamma_{a}$$

where $E_{p}$, $E_{b}$ are elastic modulus of the PZT and the host structure, respectively, and $G_{a}$ is shear modulus of the adhesive. Substituting Equations (2) and (3) into Equation (1), yields:

(4a) $$\frac{d^{2}\varepsilon_{p}}{dx^{2}}-\frac{G_{a}(\varepsilon_{p}-\varepsilon_{b})}{E_{p}h_{p}h_{a}}=0$$

(4b) $$\frac{d^{2}\varepsilon_{b}}{dx^{2}}+\frac{\alpha G_{a}(\varepsilon_{p}-\varepsilon_{b})}{E_{b}h_{b}h_{a}}=0$$

The following Equations can also be obtained as:

(5a) $$\frac{d^{4}\varepsilon_{p}}{dx^{4}}-\Gamma^{2}\frac{d^{2}\varepsilon_{p}}{dx^{2}}=0$$

(5b) $$\frac{d^{4}\varepsilon_{b}}{dx^{4}}-\Gamma^{2}\frac{d^{2}\varepsilon_{b}}{dx^{2}}=0$$

where $\Gamma^{2}=\frac{G_{a}}{E_{p}h_{p}h_{a}}(1+\frac{\alpha }{\psi })$ denotes shear lag parameter, and $\psi =\frac{E_{b}h_{b}}{E_{p}h_{p}}$.

Equation (5a,b) are decoupled equations, but $\varepsilon_{p}$ and $\varepsilon_{b}$ are related as shown in Equation (4). $\varepsilon_{p}$ and $\varepsilon_{b}$ can be obtained by solving Equation (5a,b):

(6a) $$\varepsilon_{p}=B_{1}+B_{2}x-\frac{\psi }{\alpha }B_{3}\sinh\Gamma x-\frac{\psi }{\alpha }B_{4}\cosh\Gamma x$$

(6b) $$\varepsilon_{b}=B_{1}+B_{2}x+B_{3}\sinh\Gamma x+B_{4}\cosh\Gamma x$$

where the coordinate origin of *x*-axis is the core of the PZT, and the positive direction of *x*-axis is right along the PZT radius. Through the third assumption, if the length of PZT is 2*l*, the boundary condition can be written as:

(7a) $$x=-l:\varepsilon_{p}=0,\varepsilon_{b}=\varepsilon$$

(7b) $$x=l:\varepsilon_{p}=0,\varepsilon_{b}=\varepsilon$$

Substituting Equation (7a,b) into Equation (6a,b), it yields:

(8) $$B_{1}=\frac{\psi }{\psi +\alpha }\varepsilon ,B_{2}=0,B_{3}=0,B_{4}=\frac{\alpha }{(\psi +\alpha )\cosh\Gamma l}\varepsilon$$

The strain of the PZT, the adhesive and the host structure can be expressed as:

(9a) $$\varepsilon_{p}=\frac{\psi }{\psi +\alpha }\left[1-\frac{\cosh\Gamma x}{\cosh\Gamma l}\right]\varepsilon =T_{\varepsilon }\varepsilon$$

(9b) $$\gamma_{a}=-E_{p}h_{p}\Gamma \frac{\psi }{\psi +\alpha }\frac{\sinh\Gamma x}{\cosh\Gamma l}\varepsilon$$

(9c) $$\varepsilon_{b}=\frac{\psi }{\psi +\alpha }\left[1+\frac{\alpha }{\psi }\frac{\cosh\Gamma x}{\cosh\Gamma l}\right]\varepsilon$$

where ε is the strain of the host structure, $T_{\varepsilon }$ represents the strain transfer coefficient from the host structure to the PZT:

(10) $$T_{\varepsilon }=\frac{\psi }{\psi +\alpha }\left[1-\frac{\cosh\Gamma x}{\cosh\Gamma l}\right]$$

By analyzing the strain transmission among the host structure, adhesive and PZT sensor, the expression of strain transfer efficiency is obtained. Then specific parameters can be brought into the quantitative analysis for the cases of strain transfer.

## 3. The Effect of Parameters on Strain Transfer Coefficient

In this section, both the theoretical model and finite element analysis (FEA) using ABAQUS are employed to analyze the effect of parameters of PZT and adhesive on strain transfer. In the application of guided wave-based SHM, PZT disks are often employed and expected to generate uniform guided wave along each in-plane direction. As a result, a PZT disk is utilized to investigate the high strain enduring ability in the FEA simulation and experiments.

### 3.1. Strain Distribution on the PZT

**Figure 3:**
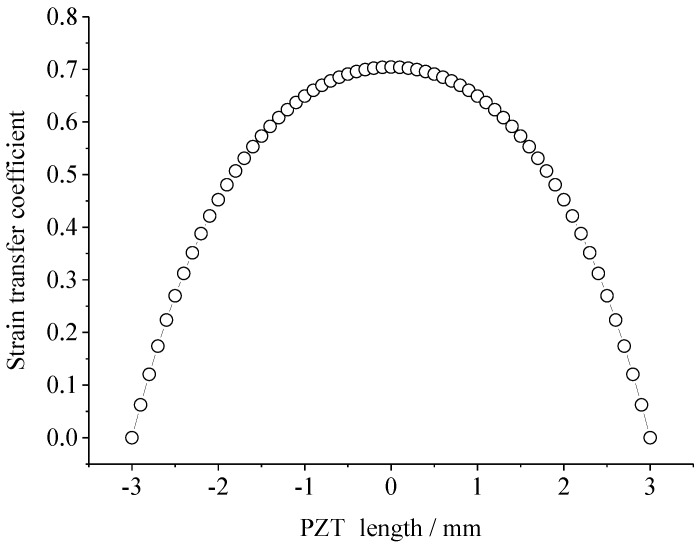
The distribution of strain transfer coefficient of PZT calculated by the theoretical model.

In the theoretical model, a one-dimensional PZT was bonded on a one-dimension host structure through adhesive. The PZT material is APC851, with an elastic modulus of 63 GPa, thickness of 0.25 mm and length of 6 mm, respectively. The material of the host structure is aluminum with an elastic modulus of 70 GPa, and a thickness of 2 mm. When the shear modulus of adhesive is 1000 MPa and the thickness is 125 μm. Parameter α is set as 1. Figure 3 shows the distribution of strain transfer coefficient of the PZT along the length. It is obvious that the strain transfer efficiency of the central region is high, and the location of the largest strain on PZT is at the center of PZT (*x* = 0). Therefore, the PZT sensor installed in the high strain area will break down from the middle part of the sensor first, and then gradually expand to the whole sensor.

**Figure 4:**
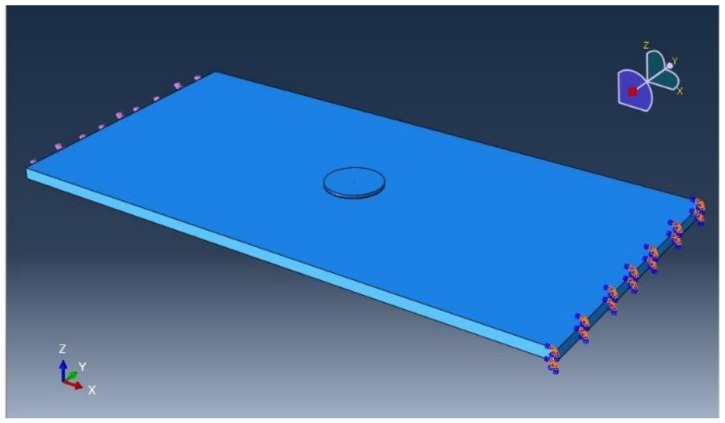
Sketch of the FEA model.

**Figure 5:**
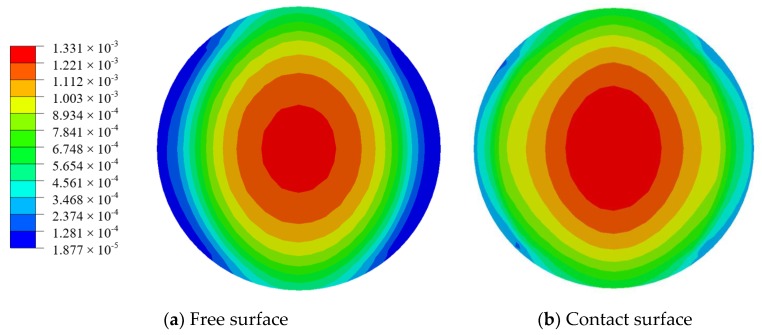
The strain distribution of PZT calculated by FEA when the strain of host structure is 2000 με.

In the FEA, as shown in Figure 4, a circular PZT is considered bonded on the surface center of the host structure, two of whose ends are uniformly loaded and the others are free. The host structure is aluminum plate with dimensions of 100 mm × 50 mm × 2 mm and elastic modulus of 70 GPa and Poisson ratio of 0.3. The shear modulus of the adhesive is 1000 MPa and the thickness is 125 μm. The PZT material is APC851 and its diameter and thickness are 6 mm and 0.25 mm, respectively. Figure 5 shows the strain distribution of two surfaces of PZT when the strain of host structure is 2000 με. It can be obviously seen that the extensive strain of the PZT center is largest, which is consistent with the results of the theoretical model.

### 3.2. The Effect of Adhesive on Strain Transfer

**Figure 6:**
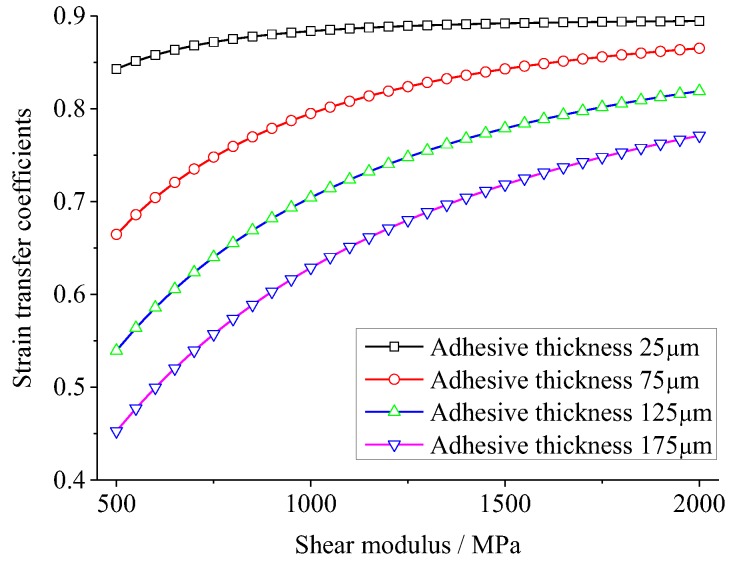
The strain transfer coefficient versus the shear modulus of adhesive in the theoretical model.

**Figure 7:**
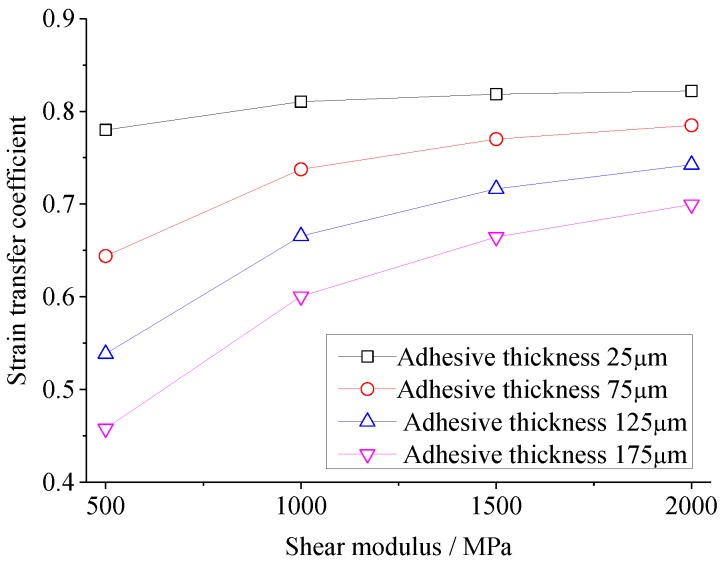
The strain transfer coefficient versus the shear modulus of adhesive in the FEA model.

The effects of shear modulus and thickness of adhesive on the strain transfer are investigated in this section. Considering the theoretical model and FEA model above, strain transfer coefficients are calculated with variations of the shear modulus of the adhesive from 500 MPa to 2000 MPa (in steps of 50 MPa of the theoretical model and 500 MPa of the FEA model), while the thickness of the adhesive is 25, 75, 125, and 175 μm, respectively. Figure 6 and Figure 7 show the strain transfer coefficients (the ratio between the strain of the PZT center and the host structure) versus the shear modulus of adhesive in the theoretical and FEM models, respectively. It can be seen that the strain transfer coefficient increases with the shear modulus growing, and the variation gradient becomes much larger as the thickness grows, while it reduces with the adhesive thickness growing, and the variation gradient becomes much smaller as the shear modulus grows. Among the range of shear modulus and thickness of adhesive, for the theoretical solution, the maximum strain transfer coefficient reaches to 89.4% at the shear modulus 2000 MPa and thickness 25 μm, while the minimum is 45.3% at the shear modulus 500 MPa and thickness 175 μm. 44.1% of strain is reduced by changing the shear modulus and thickness of adhesive for the theoretical solution, while the reduction in the FEA is 36.4%. 

**Figure 8:**
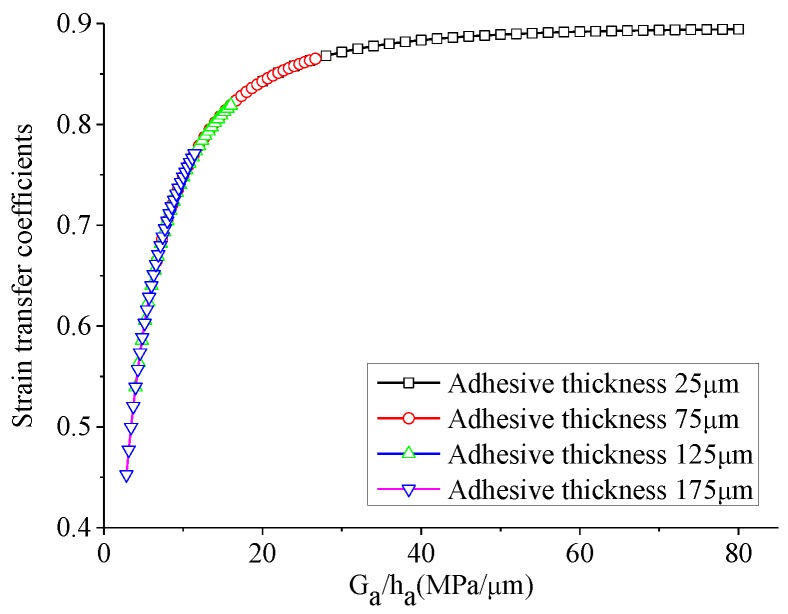
The strain transfer coefficient at the middle of PZT versus the shear modulus of adhesive in the theoretical model.

It can be inferred from Figure 6 and Figure 7 that at the different adhesive thickness, the strain transfer coefficients versus the shear modulus of adhesive have the similar shapes, except the values of the strain coefficients in Figure 6 and Figure 7 are a bit different due to the geometry differences between 1D theoretical model and 3D FEA model. If changing the horizontal coordinate of Figure 6 from the shear modulus G_a_ to the ratio between the shear modulus and thickness of adhesive, i.e., G_a_/h_a_, an interesting curve connecting four curves in Figure 6 occurs, as shown in Figure 8. It can be concluded that strain transfer coefficient is related to G_a_/h_a_. In fact, it can be obviously seen from Equation (10) that when material property of adhesive changes, only shear lag parameter Γ alters so as to affect the strain transfer coefficient. The square of Γ is proportional to G_a_/h_a_. It also can be concluded that the limitation of the strain transfer coefficient is equal to ψ/(ψ+α) when G_a_/h_a_ approaches to infinite. In this simulation, this limitation is 89.6%. 

### 3.3. The Effect of Geometries of PZT on Strain Transfer

**Figure 9:**
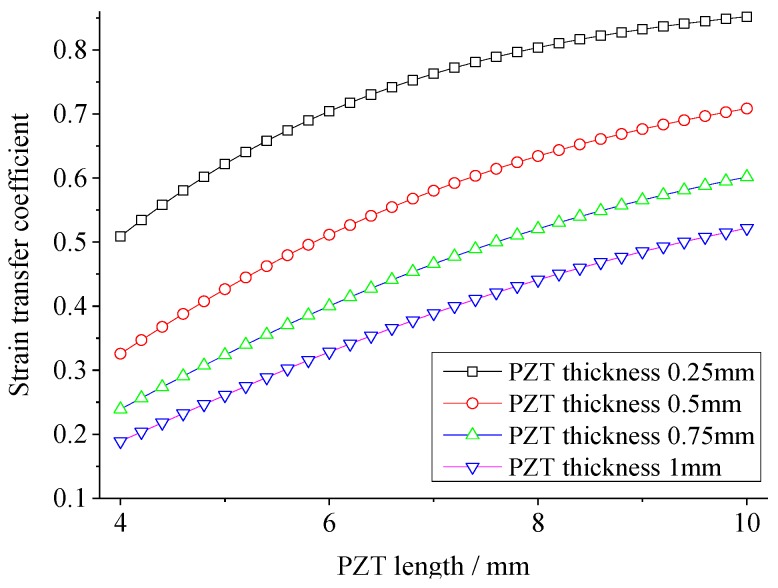
The strain transfer coefficient versus the PZT length in the theoretical model.

**Figure 10:**
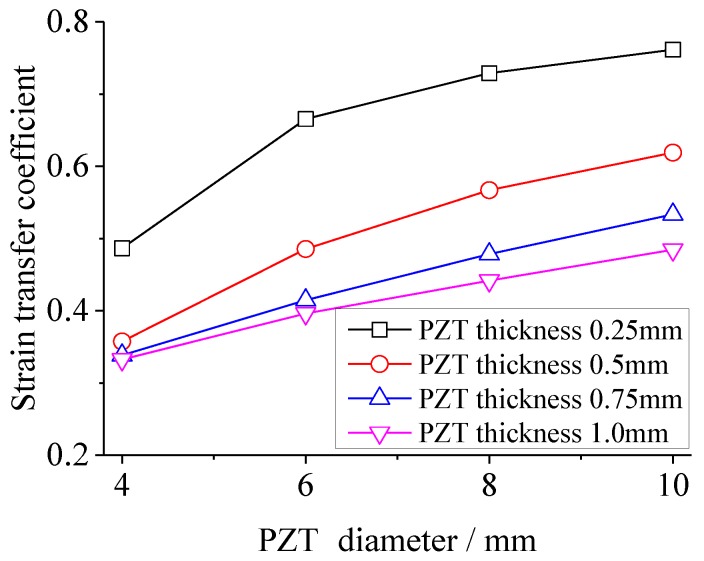
The strain transfer coefficient versus the PZT diameter in the FEA model.

Strain transfer coefficients are calculated with variations of the length/diameter of PZT from 4 mm to 10 mm (in steps of 0.2 mm of the theoretical model and 2 mm of the FEA model), while the PZT thickness is 0.25, 0.5, 0.75 and 1.0 mm, respectively. Figure 9 and Figure 10 give the strain transfer coefficients versus the PZT length/diameter in the theoretical and FEM models, respectively. It can be seen that the strain transfer coefficient increases with the PZT length/diameter growing, while the strain transfer coefficient decreases with the PZT thickness growing. Among the range of PZT length/diameter and thickness, for the theoretical solution, the maximum strain transfer coefficient reaches to 85.2% at the PZT length/diameter 10mm and thickness 0.25 mm, while the minimum is 18.9% at the PZT length/diameter 4mm and thickness 1 mm. 66.3% of strain is reduced by changing the PZT length/diameter and thickness for the theoretical solution, while the reduction in the FEA is 42.9%. This is because the PZT is not uniformly extended and the assumption condition of the theoretical model is not suitable again when the ratio between PZT thickness and length becomes large. However, no matter for the theoretical or FEA solution, a big reduction can be realized by changing PZT geometry. 

## 4. The Effect of PZT Configuration on Strain Transfer Coefficient

**Figure 11:**
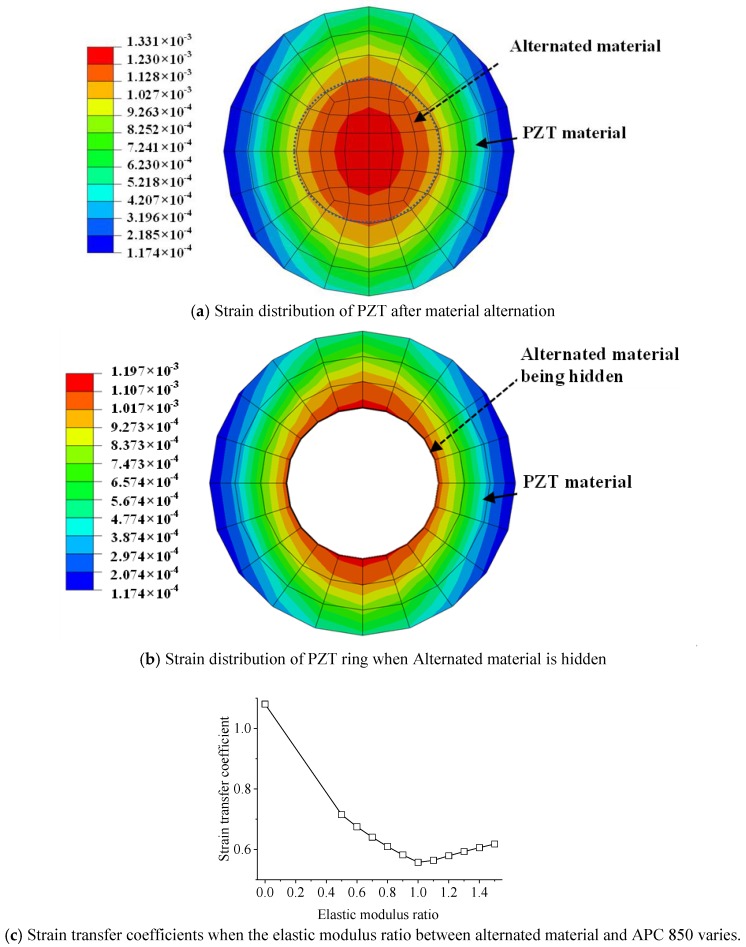
Strain distribution when material of PZT center is alternated.

Through the strain distribution of PZT disk shown in Figure 5, it is clear that the closer the location is to the center of PZT, the larger the strain is. It is feasible to alternate the material at the center of PZT as another material with good elongation to increase the survivability of PZT at high strain area. For the case in Section 3.1, consider to alter the center district of PZT with diameter 3 mm as another material. As shown in Figure 5 and Figure 11a, when material of the PZT center is altered as another material, with similar elastic modulus but better elongation property, the strain distribution after material alternation is similar as that before, and the largest strain value is close to each other.

When the elastic modulus of the altered material at the PZT center ranges from 0.5 to 1.5 times in step of 0.1 times large than the elastic modulus of PZT, it can be concluded from Figure 11c that the strain transfer coefficient enhances when the elastic modulus ration keeps away from one. It is because when the gap between elastic modulus of two materials becomes bigger, a worse and worse deformation compatibility occurs so that the strain of the PZT ring becomes larger. When the elastic modulus of central material approaches 0, i.e., the PZT ring has an empty center, the strain transfer coefficient is largest. It is clear that a material, with a good elongation and a similar elastic modulus, to alter the PZT center is preferred to reduce the strain transfer from the host structure.

## 5. Experimental Verification

An experiment was conducted to verify the correctness of the above theoretical model and finite element analysis. The PZTs were bonded on the specimen using adhesive with different thickness and shear modulus. Since electromechanical impedance method is one of the most common SHM techniques for damage detection by using PZT sensors [7,8,9,14,15,16,17], the impedance of PZT was employed to assess whether the PZT is failure or not due to the increase of the static load.

**Figure 12:**
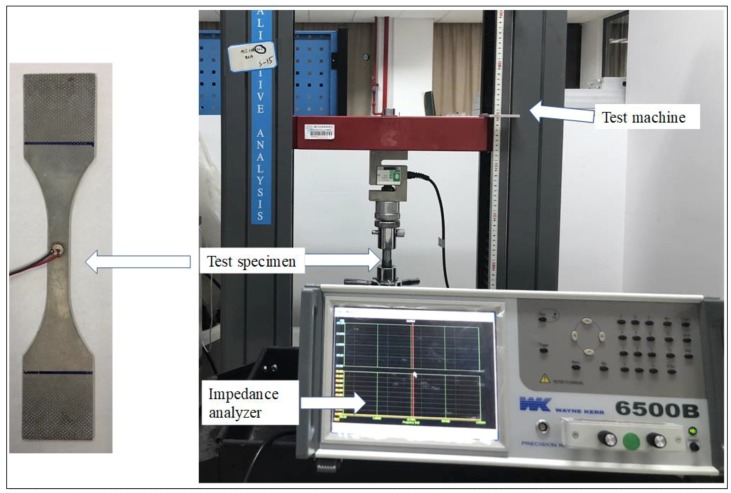
Impedance analysis of the PZT.

The PZTs used in the experiment are APC851 with a diameter of 6.35 mm and thickness of 0.25 mm. An impedance analyzer WK6500 developed by Wayner Kerr Electronics Company in London, UK was utilized to acquire the impedance of a PZT sensor bonded on the surface of an aluminum specimen by Hysol EA 9395 or Hysol EA 9396 developed by Henkel Adhesive Technologies in Westlake, OH, USA, as shown in Figure 12. A digital electro-hydraulic-servo test machine was used to applied tensile load on the aluminum specimen. Material characteristics of the adhesives are shown in Table 1.

**Figure 13:**
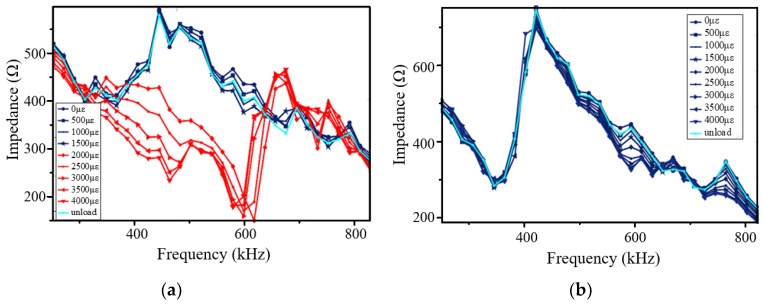
The impedance of PZT under different strain conditions at the adhesive (Hysol EA 9395) thickness (**a**) 25 μm and (**b**) 125 μm.

When Hysol EA 9395 with thickness of 25 μm was adopted as the adhesive to bond the PZT on the aluminum specimen, the impedances of PZT under different strain conditions of the aluminum plate, from 0 to 4000 με in steps of 500 με, were acquired, as shown in Figure 13a. When the strain of the aluminum plate is larger than 2000 με, the impedance of the PZT has been greatly changed among the working frequency range of guided wave, which is similar to Qing’s viewpoint that when the strain on the PZT exceeds the elongation, the PZT failure occurs [14]. When the thickness of Hysol EA 9395 was 125 μm, the impedance of the PZT is shown in Figure 13b. It can be seen that there is only a slight change among the PZT impedances under different strain conditions up to 4000 με. 

Based on Figure 7, it can be calculated that the strain transfer coefficient is just above 0.5 when the thickness of adhesive is 25 μm, while the strain transfer coefficient reduces to about 0.25 when the thickness of adhesive increases to 125 μm. Therefore, using Hysol EA 9395 with thickness of 25 μm to bond PZT, the largest strain in the PZT is above 1000 με, which is close to the elongation of PZT material, when the strain of host structure is more than 2000 με. Using Hysol EA 9395 with thickness of 125 μm to bond PZT, the largest strain in the PZT is just around 1000 με when the strain of the host structure reaches 4000 με.

**Figure 14:**
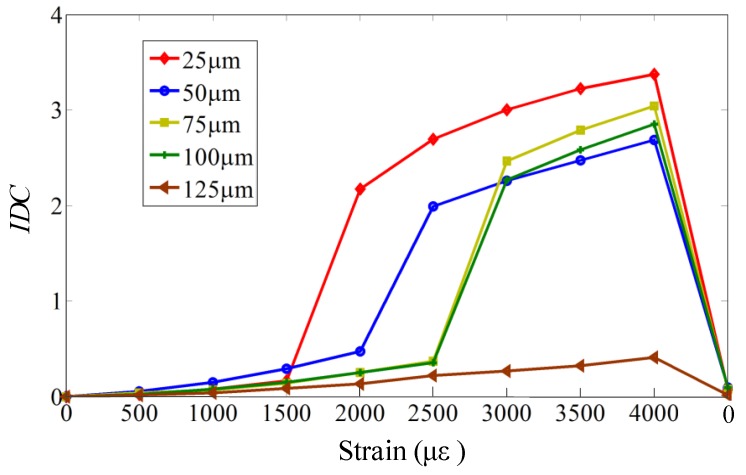
The impedance difference variation with the strain.

In order to further verify the variation trend, the impedances of PZT under different strain conditions were studied when the adhesive thickness was set as from 25 μm to 125 μm in steps of 25 μm. An impedance difference coefficient (IDC) is introduced to indicate the difference under current strain condition and free strain condition as:

(11) $$IDC=\frac{\int_{\omega_{1}}^{\omega_{2}}{\left(I_{c}\left(\omega \right)-I_{0}\left(\omega \right)\right)}^{2}d\omega }{\int_{\omega_{1}}^{\omega_{2}}{\left(I_{0}\left(\omega \right)\right)}^{2}d\omega }$$

where $I_{c}\left(\omega \right)$ and $I_{0}\left(\omega \right)$ represent the impedances under current and free strain conditions, respectively. Figure 14 gives the variation of impedance difference coefficients with the strain when the adhesive has different thickness. It can be concluded from Figure 14 that as the adhesive thickness increases, the impedance difference coefficients decrease, which may contribute to the decrease of the strain transfer from the host structure to the PZT.

**Figure 15:**
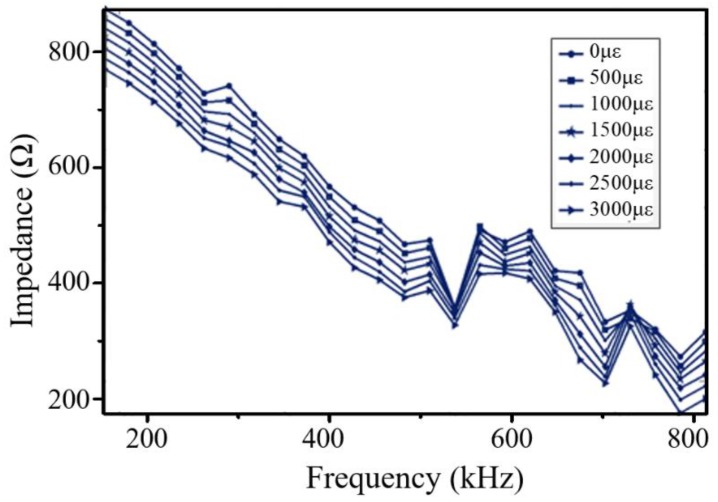
The impedance of PZT under different strain conditions at the adhesive.

Figure 15 shows the impedances of PZT under different strain conditions when Hysol EA 9396 was used as the adhesive. It can be obviously seen that the large changes caused by the high strain using Hysol EA 9395 do not occur when using Hysol EA 9396, which indicates that the strain transfer coefficient decreases as the shear modulus of the adhesive is reduced. This verifies the simulation results in Figure 7.

## 6. Discussion and Conclusions

Due to the material nature, PZT sensors have low reliability when mounted at locations exposed to a large tensile strain. A novel approach to greatly improve the strain survivability of PZT by optimal design of the bonding adhesive has been proposed. A one-dimension theoretical model is proposed and three-dimension finite element model is developed to study the strain transfer from the host structure to the PZT sensor. Although there is a slight value difference of strain transfer coefficients for the geometry difference between the theoretical model and finite element model, the results from both models show very similar variation trends of strain transfer coefficients with the adhesive parameters, and are mutual authenticated. Based on the theoretical model established, finite element method developed and experiments conducted, it is clear that the PZT sensor can work perfectly when it is mounted on the host structure with tensile strain up to 4000 με by using optimal adhesive. This is because the strain transferred to the PZT is still less than 1100 με, which is the maximum tensile strain PZT can bear, even the strain of host structure reaches 4000 με. The following remarks can be made based on the above investigation: 

With the increase of the thickness of adhesive layer, the efficiency of strain transfer decreases, the maximum strain transferred from the host structure to the PZT sensor decreases, as well as the high strain area decreases, concentrating to the center of PZT sensor.

With the increase of the shear modulus of the adhesive layer, the efficiency of strain transfer increases. After the shear modulus of adhesive layer increases to a certain value, the efficiency of strain transfer at area with maximum strain will not increase, but the high strain region increases.

With increasing radius/length of the PZT sensor, the efficiency of strain transfer increases, and the high strain area increases.Alternating the material at the center of PZT sensor with another material with good elongation can increase the survivability of PZT in high strain areas.

## Figures and Tables

**Table 1:** Material of adhesive in the experiment.

Material Name	Hysol^®^ EA 9395	Hysol^®^ EA 9396
Shear modulus (MPa)	1900	1050
Elongation at break (%)	2.6	3.4
Density (g/mL)	1.27	1.14
